# Artificial Intelligence (AI) and Internet of Medical Things (IoMT) Assisted Biomedical Systems for Intelligent Healthcare

**DOI:** 10.3390/bios12080562

**Published:** 2022-07-25

**Authors:** Pandiaraj Manickam, Siva Ananth Mariappan, Sindhu Monica Murugesan, Shekhar Hansda, Ajeet Kaushik, Ravikumar Shinde, S. P. Thipperudraswamy

**Affiliations:** 1 Electrodics and Electrocatalysis Division, CSIR-Central Electrochemical Research Institute (CECRI), Karaikudi, Sivagangai 630003, Tamil Nadu, India; siva.cecri21a@acsir.res.in (S.A.M.); sindhumonicam98@gmail.com (S.M.M.); 2 Academy of Scientific & Innovative Research (AcSIR), Ghaziabad 201002, Uttar Pradesh, India; shansda@cecri.res.in (S.H.); tpswamy@cecri.res.in (S.P.T.); 3 Corrosion and Materials Protection Division, CSIR-Central Electrochemical Research Institute (CECRI), Karaikudi, Sivagangai 630003, Tamil Nadu, India; 4 School of Engineering, University of Petroleum and Energy Studies (UPES), Dehradun 248001, Uttarakhand, India; akaushik@floridapoly.edu; 5 NanoBioTech Laboratory, Department of Environmental Engineering, Florida Polytechnic University, Lakeland, FL 33805-8531, USA; 6 Department of Zoology, Shri Pundlik Maharaj Mahavidyalaya Nandura, Buldana 443404, Maharashtra, India; ravikumar.shinde@gmail.com; 7 Central Instrument Facility, CSIR-Central Electrochemical Research Institute, Karaikudi, Sivagangai 630003, Tamil Nadu, India

**Keywords:** artificial intelligence, Internet of Medical Things, healthcare, smart sensors, wearable devices, point-of-care sensors

## Abstract

Artificial intelligence (AI) is a modern approach based on computer science that develops programs and algorithms to make devices intelligent and efficient for performing tasks that usually require skilled human intelligence. AI involves various subsets, including machine learning (ML), deep learning (DL), conventional neural networks, fuzzy logic, and speech recognition, with unique capabilities and functionalities that can improve the performances of modern medical sciences. Such intelligent systems simplify human intervention in clinical diagnosis, medical imaging, and decision-making ability. In the same era, the Internet of Medical Things (IoMT) emerges as a next-generation bio-analytical tool that combines network-linked biomedical devices with a software application for advancing human health. In this review, we discuss the importance of AI in improving the capabilities of IoMT and point-of-care (POC) devices used in advanced healthcare sectors such as cardiac measurement, cancer diagnosis, and diabetes management. The role of AI in supporting advanced robotic surgeries developed for advanced biomedical applications is also discussed in this article. The position and importance of AI in improving the functionality, detection accuracy, decision-making ability of IoMT devices, and evaluation of associated risks assessment is discussed carefully and critically in this review. This review also encompasses the technological and engineering challenges and prospects for AI-based cloud-integrated personalized IoMT devices for designing efficient POC biomedical systems suitable for next-generation intelligent healthcare.

## 1. Introduction

**Figure 1:**
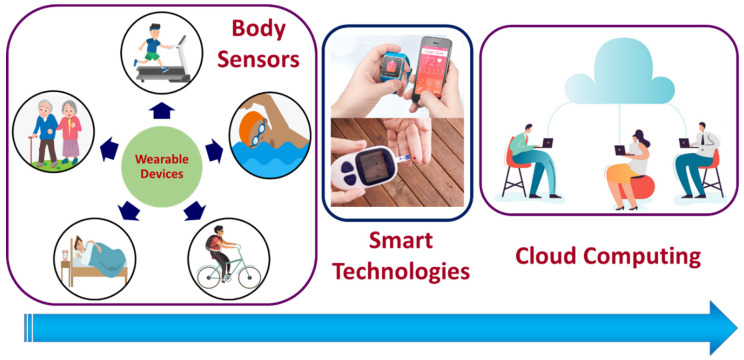
Schematic representation of IoMT devices and cloud data transfer. Body sensors are those that are directly attached to the body, embedded in fabric, or implanted into the human body. Smart sensing technology is used to analyze the collected data and transfer it to the cloud. The cloud serves as a bridge between body sensors and the recipient of the output.

The Internet of Medical Things (IoMT) is the subsets of the Internet of Things (IoT) technologies that consists of inter-network-connected medical devices for healthcare monitoring. IoMT devices, also referred to as healthcare IoT, enable human intervention-free healthcare monitoring by integrating automation, interfacial sensors, and machine learning-based artificial intelligence. IoMT technology connects patients with clinicians through medical devices, allowing remote access to collect, process, and transmit medical data over a secured network. IoMT technologies aid in reducing unnecessary hospital stays and thereby the associated health costs by facilitating wireless monitoring of health parameters. The IoMT medical technologies segment covers wearable, in-home personal real-time health-monitoring devices and hospital or clinical-based point-of-care (POC) devices [1]. The wearable personal health monitoring device category includes smart wristbands, electronic textiles and garments, smartphone-integrated devices, and sports watches for fitness and activity monitoring [2], as illustrated in Figure 1.

IoMT-enabled POC monitoring for clinical applications includes an on-demand medical examination and the tele-visit system “TyroPro” (https://www.tytocare.com/professionals/, assessed on 22 July 2022). IoMT wearable devices can also monitor involuntary falls of older adults. Falls in the older population are inevitable, but their circumstances can be monitored and prevented to avoid chronic injuries. IoMT technologies aid in reducing unnecessary hospital stays and thereby the associated health costs by facilitating wireless monitoring of health parameters.

Traditional healthcare monitoring is undergoing transformational change, and digital healthcare enables automated detection tools and integrated cloud products in consumers’ hands. This digital transformation enables patients, clinicians, and people in rural communities to access quality healthcare services for a better outcome. POC devices such as ultrasound, thermometers, glucometers, and ECG readers come with Internet connectivity and cloud storage facilities that let users track their health. The improvements in these technologies are crucial for improvised healthcare for adjusting insulin doses and connecting the patients directly with clinicians. Advanced healthcare centers have started utilizing the smart-bed concept, which could alter the bed’s angle and position by monitoring the patient’s sleeping posture. IoMT-enabled devices also help transform traditional home healthcare services. For example, the intelligent home-medicine dispensing system automatically uploads information about patient medical history to the cloud. It alerts doctors and patients about the medication that must be taken and alerts the clinician when the patient is not taking medicine. Technological advancement, industrial adaptation, and urbanization are increasing demands in the healthcare system with the rising population. The improvement in IoMT-incorporated devices with smartphones, sensors, and actuators can ensure periodic healthcare monitoring, and all these aspects are the focus of this review.

## 2. The Role of AI in Establishing a Smart Sensor Network

Artificial intelligence (AI) has been a topic of interest among researchers and biomedical industries due to its ability to process large amounts of data, produce accurate results, and control processes to generate the most optimized outcome. AI is not new because machines are being used for decision-making and predicting the expected effects of diseases and consequences in the longer term. In this modern world, most day-to-day tasks are assisted by machines and algorithms. Several factors, such as fairness, explainability, accountability, reliability, and acceptance, are considered using reliable machine–algorithm-coordinated outcomes [3]. AI can be interpreted as the ability of the computer or robot to reproduce human intelligence in the form of software and algorithms. AI can perform intellectual processes such as logical reasoning, knowledge-based learning, drug discovery, guided surgery, and advanced imaging. The recent emerging interest in AI may be new, but this concept was already established in the late 1940s by various researchers [4,5,6], and their ideas are still valuable as the foundation of recent AI-based investigations and inventions. In 1972, researchers in Japan built the world’s first humanoid robot, “WABOT-1”, which can communicate with a person in Japanese and measure distances and directions. However, due to computing power and funding limitations, AI research faced stagnation until the late 1990s. During the late 1990s, big tech companies like IBM started working on AI-based models. In the mid-2000s, social network platforms, email service providers, search engines, and many other companies that process a large amount of data benefited from AI models and programs. One of the reasons for the recent expansion in AI is the improvement in computational power in CPUs and the applicability of GPUs in the field of computations. The other reason for adopting an AI-based system is the big data created by the user demand needed for better analytics.

**Figure 2:**
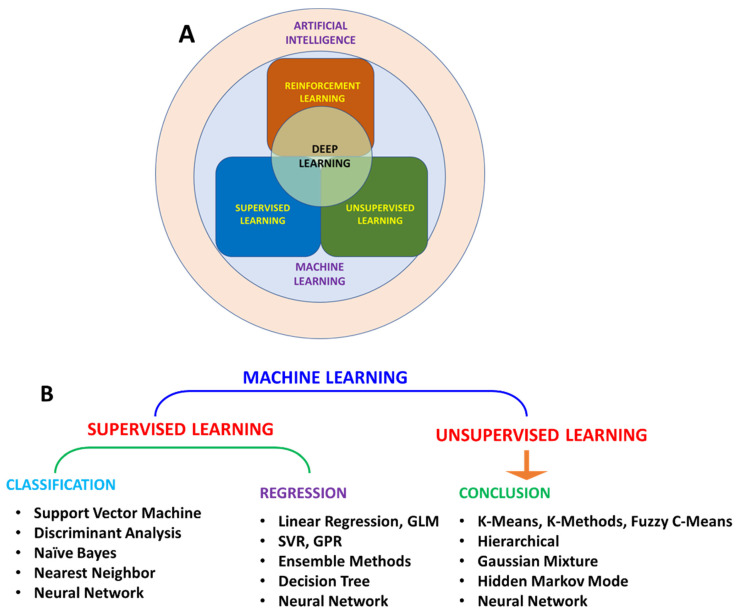
Schematic representation of relation between AI, ML, and DL (**A**); classification of ML algorithm (**B**).

Machine learning (ML) is the most widely used AI method for making predictions from patterns (Figure 2A). Based on the algorithm structure and learning method, ML can be further classified into various types (Figure 2B). Learning methods can be further classified as supervised, unsupervised, and reinforced learning. In supervised learning, the algorithm is trained with input data. Supervised learning is used in applications where historical data are available and can be used to predict possible future events. As these algorithms use historical data for training, methods are more straightforward and accurate. These algorithms can be further divided into regression and classification algorithms. Regression algorithms can be used when the input variable and output variable have a relationship, such as in weather forecasting. In classification algorithms, output variables can be categorized into classes such as yes–no and true–false with respect to input variables. Due to these features, supervised learning can solve a real-world problem and predict the output based on available data. Unsupervised learning methods can identify a pattern in each dataset even if data are not classified or labelled correctly.

In this direction, algorithms become computationally complex, and accuracy also decreases. These algorithms can divide data into groups based on similarities and differences in data. These can be divided into two categories: (a) clustering and (b) association. In the clustering algorithms group, the data in clusters based on similarities, such as the purchase behavior of a group of customers, helps in marketing products. An association algorithm is a rule-learning algorithm that finds relationships in variables in each dataset, for example, shopping patterns of individual customers to suggest products to customers. Reinforced learning is a reward/penalty-based learning method. Algorithms assign positive values to desired results and negative values to undesired effects. These algorithms are hard to train and time-consuming. Deep learning (DL) is a particular type of ML that teaches computers to mimic human behavior. DL uses neural networks (NN), which require much computational power for a complex problem. However, recent computing power and data analytics advancements have enabled DL algorithms to observe, learn, and react to difficult situations. The DL algorithm can adopt supervised, unsupervised, or reinforced learning approaches based on the desired application. The best application example is email provider services, where AI is used to separate spam from essential emails, and with each new datum, its accuracy improves. Most of the current weather forecasts are based on AI model prediction.

AI-based models have shown their worth in pharmaceutical and healthcare industries by improving the efficiency in therapeutic drug manufacturing, real-time health monitoring, and predictive forecasting. AI has already shown its promise in drug discovery and is being implemented in different phases, from drug design to drug screening [7,8,9,10,11]. In 2020, the DL model “Alphafold” solved a 50-year-old problem by accurately predicting the structure of a protein from its amino acid sequence [9,10,11]. Alphafold performed better with 0.7 and higher TM scores for 24 out of 43 free modeling domains compared to the second best protein-structure prediction method, which achieved such accuracy for only 14 out of 43 domains in a blind assessment [10]. AI has proven to be a potential tool in the early-stage detection of Alzheimer’s [12], cancer [13], diabetes [14], and cardiac diseases, even in the asymptomatic stages. There are applications for which AI is already in use by health industries. The most widely used application of AI in healthcare is the management of medical records and patient history. These data can be used by digital consultation apps, like Babylon in the UK and Buoy Health in the USA, which require a symptoms list, patient history data, and common medical knowledge to diagnose and offer a recommended action.

**Figure 3:**
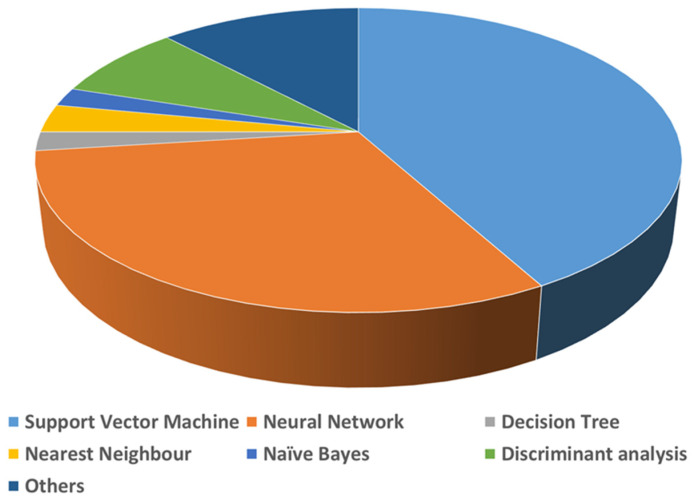
Use of various AI methods in medical applications.

A list of detailed uses of various AI algorithms and learning methods in the medical literature was published by Jiang et al. [15]. According to this review, supervised learning is the recommended learning method for healthcare, as it provides more clinically relevant results. Jiang et al. further reported that support vector machines (SVMs) and neural networks (NNs) are the primarily used AI-based algorithms for medical applications (Figure 3). A brief comparison of the applications along with the advantages and disadvantages of SVMs, NNs and other common AI algorithms used in biomedical applications are given in Table 1. Natural language processing (NLP) is another field in AI required for full integration of AI for real-world applications. NLP enables machines/computers to understand, analyze, manipulate, and potentially generate human language. It takes input as written or spoken text. Coupling NLP and ML algorithms can enable them to do complex tasks. Common examples of this category are virtual assistants such as Google Assist, Siri, and Alexa. NLP is also used for automated encoding of clinical documents [16]. Recently, during COVID-19, NLP methods have been put in practice to process clinical notes into a machine-readable format, which helps in highlighting the patient’s condition and medical history, subjective assessment results, and the advice provided to them [17].

AI-based systems are also customizing the most effective treatment path along with precise medications for individuals based on medical records and patient history. Wearable health tracker devices can easily monitor and provide data on patients’ heart rate and activity levels to health services. As the amount of data is enormous and coming from many sources, AI-based solutions are used to process data and find anomalies for individuals. Similarly, in hospitals, data generated from health-monitoring devices for individual patients can find possible emergencies and alert health workers. Some countries, like Norway and Denmark, are already using healthcare system analysis to highlight treatment mistakes and workflow inefficiencies. This way, AI is helping by reducing the burden of the healthcare system, avoiding wrong diagnoses and unnecessary patient hospitalizations, and saving money and time for patients by avoiding unnecessary appointments. The knowledge-based training of DL models requires input datasets obtained from the clinical trial data. For example, functional magnetic resonance imaging (fMRI) data collected from Alzheimer’s patients and lung cancer computerized tomography (CT) scans are used as input files for AI-assisted diagnosis of Alzheimer’s and lung cancer, respectively. The subject-level classification for 138 subjects for associated stages of AD resulted in an accuracy of 100% for cognitively normal (CN), 96.85% for subjective memory complaints (SMC), 97.38% for early mild cognitive impairment (EMCI), 97.43% for late mild cognitive impairment (LMCI), 97.40% for mild cognitive impairment (MCI), and 98.01% for AD [35]. The input data collected over the years also helps identify the patterns in the data. A DL model developed by Etemadi et al. based on 40,000 earlier available CT scans outperformed veteran radiologists by identifying earlier lung cancer with 94% accuracy. [31]. These approaches will help in the early-stage detection of lung cancers, which is extremely important in healthcare because ~70% of lung cancers are detected in later stages, resulting in a low survival rate [13].

Similarly, DL models have been developed for analyzing breast cancer [51,52] and pancreatic cancer [32,53]. The DL model developed by Alexander et al. achieved a sensitivity of 50.9% as compared to 22.4% of the commonly used method based on breast density. The DL model developed by Alexander et al. achieved a sensitivity of 50.9% compared to 22.4% of the commonly used method based on breast density. Muhammed et al. and Qureshi et al. developed DL models based on CT scan data, giving an accuracy of 80% [32] and 86% [53], respectively. There are similar examples of the prediction of heart failure by AI using electrocardiogram (ECG) data [54,55]. Using only the ECG data as input for the DL model, Akbilgic et al. achieved an area under the receiver operating characteristic curve (AUC) of 0.756 (0.717–0.795), which showed a further improved AUC of 0.818 (0.778–0.859) when using ECG-AI model output, age, gender, race, body mass index, smoking status, prevalent coronary heart disease, diabetes mellitus, systolic blood pressure, and heart rate as predictors [54]. Another DL model developed by Bagci et al. achieved 95% accuracy in finding specks of cancer in CT scans as compared to the 65% of average accuracy rate of radiologists [30]. The developed DL model assisted in detecting lung cancer, wherein the CT scan data failed to find any abnormalities. This approach will help in the early-stage detection of lung cancers [13], which is extremely important in healthcare because ~70% of lung cancers are detected in later stages, resulting in a low survival rate. DL models for analyzing pancreatic cancer are also developed by investigating CT scan images and other related clinical data. There are similar examples of the prediction of heart failure by AI using electrocardiogram (ECG) data [13]. Adopting AI in robotic surgeries, especially spinal surgery, is also a point of interest for healthcare industries. AI-based robots can analyze data from previous surgical procedures to develop new surgical methods. These robots can perform surgery more accurately with reduced accidental movements [25]. Apart from spinal surgery, AI also finds applications in minimally invasive surgery, surgeries assisted by robots, and post-surgery care, such as calculating recovery time [56].

**Figure 4:**
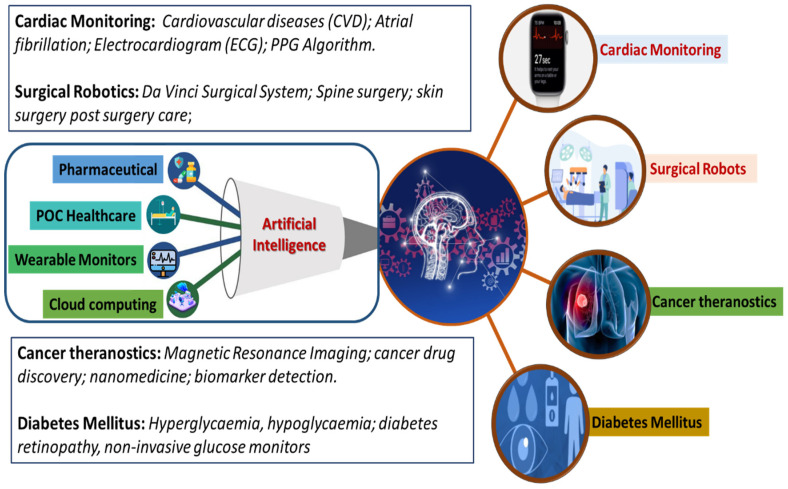
Schematic representation of the role of AI-based approaches in various themes of healthcare research, including cardiac monitoring, surgery, cancer theragnostic, and diabetes mellitus management.

The commercialization of developed AI-based systems has established a technology transformation platform called “PathAI”. Such AI-based technologies have proven to improve patient’s health outcomes via efficient pathological diagnosis. Similarly, *“PAGER”* is a healthcare management application helping with patient treatments by giving appropriate recommendations. AI also benefits the drug-development sector by finding possible new drugs by using molecular modeling and training medical data. AI has also helped in the development of technologies for human–machine interfaces. Technologies involving human–machine interfaces require a sensor that generates high-quality data and an AI algorithm with powerful data analysis capability. These have been found to be helpful in the medical field. A few reported examples are artificial limbs and wearable sensors to collect real-time data about the patient [19,57,58]. This review focuses on exploring advancements in developing IoMT and AI-assisted platforms for cardiac monitoring, cancer diagnosing, surgeries, diabetic monitoring, and other related diseases, as illustrated in Figure 4.

## 3. Role of Nanotechnology and IoMT in Healthcare

**Figure 5:**
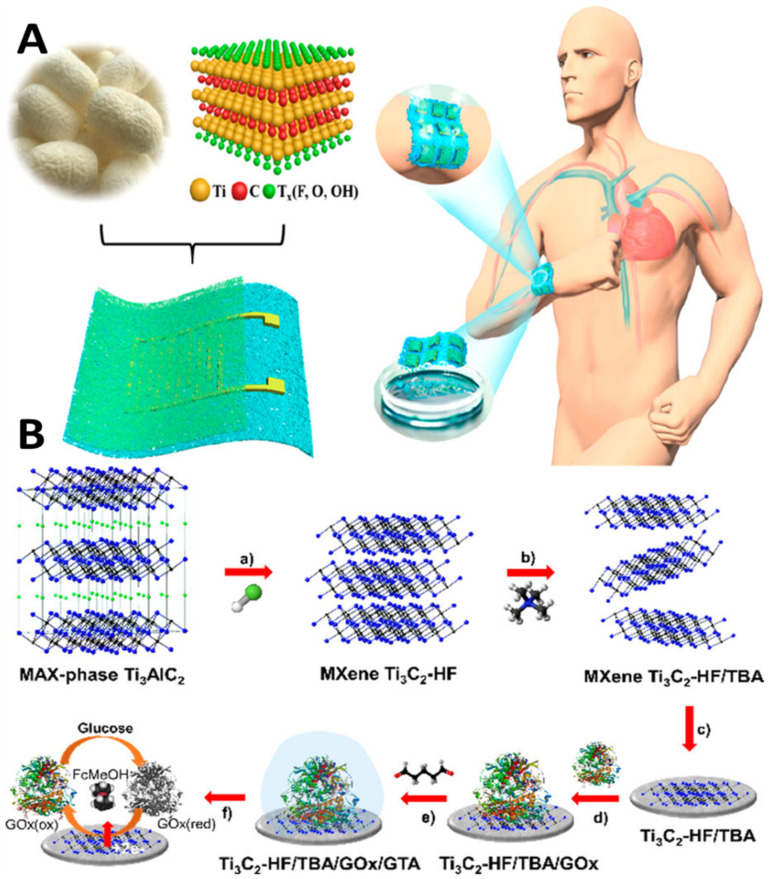
Mxene as a breathable and biodegradable material for developing E-skin-based pressure sensors (**A**) [63]. Utilizing the HET and high surface area of Mxene for developing second-generation glucose-monitoring devices (**B**) [64]. (Reproduced with permission from the American Chemical Society).

Innovations and inventions in the biomedical field help enhance health care quality. However, the role of nanotechnology needs a better understanding, and more profound knowledge through AI is crucial for desired sensitivity and miniaturization. Bridging the link between nanotechnology and AI-assisted systems interfaced with IoMT technology is critical for developing innovative healthcare solutions, including nanomedicine and nanorobotics. The applications of nanotechnology-enabled wearable continuous-monitoring devices are popular in the healthcare sector. Advanced wearable devices are nano-enabled sensor embedded, enabling the device to monitor physiological parameters continuously. An extensive clinical data set requires AI analysis and training for accurate diagnosis and prognosis [59]. The miniaturized devices interlink a broad category of research areas, including nanotechnology and biomedical engineering, to circumvent the challenges in diagnosis and therapeutics (surgery and targeted drug therapy) [60]. The introduction of advanced two-dimensional (2D) functional materials such as graphene, borophene, and MXenes has enabled the generation of next-generation bio-sensing devices with improved spatiotemporal features [61,62]. Recently, MXene-integrated e-skin-based sensors for monitoring human motions based on the pressure transduction principle have also been reported [63] (Figure 5A).

**Figure 6:**
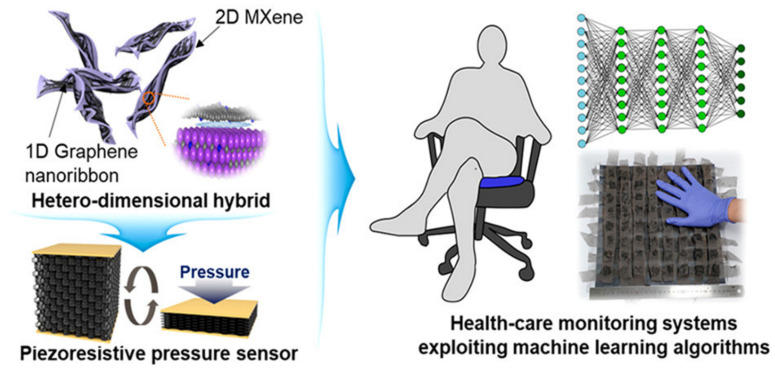
Interfacing interconnection of 1D graphene nanoribbons with 2D Mxene for developing pressure sensors trained using a machine learning algorithm. (Reproduced with permission from the American Chemical Society [66]).

The biodegradable Mxene pressure sensor demonstrated excellent breathability. The sensor can be interfaced with wireless smart sensing devices for practical applications, including human locomotion monitoring, biodegradable implanted devices, intelligent electronic skins, and therapeutic monitoring. MXene has evolved as excellent interfacing material in healthcare and environmental gas sensing with remarkable sensitivity and selectivity. In the case of glucose monitoring, the electrical properties and inherent heterogeneous electron transfer (HET) characteristics of MXene have been investigated (Figure 5B) for developing second-generation glucose-sensing devices [64]. Direct and rapid blood-glucose measurement is crucial for managing diabetes mellitus in a personalized manner. The physicochemical properties of Mxene help in enhancing the sensitivity of the sensing devices for biomedical, environmental, and food analytics applications [65]. Creating hybrid nanocomposite materials by combining a 2D Mxene with 1D nanostructures for enhancing the adhesion stability on the transducer surface for long-term monitoring has also been attempted [66]. The interfacial integration of a 2D Mxene/1D graphene nanoribbon has been investigated for developing the desired pressure sensor with an improved life cycle. ML approaches were utilized for training the sensors for detecting various sitting postures with >95% accuracy (Figure 6). Sharma et al. recently highlighted the importance of 2D borophene systems supported by IoMT smartphone-supported high-performance applications [67].

Carbon-based nanomaterials such as carbon nanotubes and graphene conserve intrinsic electrical properties and excellent biocompatible properties required for bio-signal monitoring. These materials allow for integration with skin-compatible devices to create wearable monitoring devices [68]. Graphene is one of the most widely studied 2D nanomaterials adopted for biomedical research due to its enhanced stability, electronic mobility, and electrical conductivity. Graphene has demonstrated a potential application for fabricating biomedical sensing devices, including optoelectronics and wearable devices. Biomimetic sensors imitating the sensory functions of the human brain, such as touch, smell, taste, and hearing, have been developed using graphene-based materials [69]. The high surface area of graphene allows the construction of biosensing devices with excellent sensitivity. On the other hand, the mechanical properties of graphene enable the design and development of wearable devices for continuous monitoring. The electrical conductivity and strength of graphene enable the devices to have a faster response time and maximum sensing range.

Strain sensors constructed using graphene mimic human fingers for tactile sensing [36]. Machine intelligence training allows the tactile sensor to improve its accuracy by over 80% in identifying surface structures and material species. The electronic properties of graphene enable the construction of electronic nose (e-nose) sensors for developing gas sensors that mimic the human olfactory system. Hayaska et al. developed an artificial olfactory system (e-nose) for detecting volatile organic gases using graphene-based field-effect transistors (GFETs) [37]. Supervised ML algorithms were used to analyze the gas-sensing patterns to improve detection accuracy. Kwon et al. reported the development of flexible printed sensors for wireless bioelectronic signal monitoring [39]. A new additive manufacturing technology uses nanomaterials such as graphene, silver, and polyimide to fabricate flexible, stretchable printed sensors for monitoring EMG signals of the skin. The skin-formal sensor does not require external gels or adhesive tapes for biopotential signal acquisition. A DL algorithm based on the CNN network has been developed for classifying the EMG signals acquired during various muscle activities.

Integrating flexible printed bio-signal-monitoring devices with AI modules could allow real-time and wireless measurement. Similar to flexible devices, electronic textile (e-textile)-based sensor platforms are also widely used for real-time and continuous measurement of physiological signals [70]. Fang et al. demonstrated an e-textile-based triboelectric pulse sensor for non-invasive blood-pressure measurement [71]. The durable and skin-conformable e-textile sensor is integrated with a triboelectric carbon nanotube (CNT) network with electrostatic induction, which converts biochemical pressure signals into measurable electricity. The CNT network provided the textile platform with excellent stability and conductivity. A customized mobile phone application (app) was also designed for real-time measurement of cardiovascular conditions. The app can process the data using peak search and calibration algorithms to improve the precession. The app can also transmit the data wirelessly to the cloud database for long-term monitoring. Gold nanoparticle (GNP) array-based artificially intelligent chemiresistive volatile gas-monitoring sensor devices have been designed for detecting preeclampsia, a hypertensive disorder during pregnancy [72]. Microelectronic devices consist of pairs of circular interdigitated electrodes coated with a GNP array used as a transducer. The intelligent GNP array responds to volatile gases due to the changes in the medium surrounding the nanoparticles. Discriminant factor analysis (DFA) algorithms were used to process the breath signal patterns and discriminate between women with preeclampsia and those with non-preeclamptic pregnancy. The developed intelligent GNP nanoarray system allows rapid measurement (~10 min) with good sensitivity, selectivity, and accuracy.

Metal–organic frameworks (MOF) are an essential class of crystalline materials constructed from inorganic metallic clusters and organic ligands. MOFs are characterized by their organized porous structures, high surface area, and vast structural diversity. The porosity of the MOFs can be tuned by selecting appropriate metal ions and suitable organic ligands. The unique physical and chemical properties make MOFs an exciting candidate for creating sensors and energy-storage devices [73]. The high surface area and porosity allow the MOF structures to load a high concentration of bioreceptor molecules such as enzymes, antibodies, and aptamers for electrochemical sensing applications. Covalent organic frameworks (COFs) belong to the family of porous coordination polymers (PCPs). Like MOFs, COFs also form highly ordered conjugated polymer networks in 2D and 3D structures. MOFs are intrinsically transparent to visible light. By harnessing the optical properties of MOFs, transparent electronics for monitoring in a gaseous environment are developed. The sensitivity of the system was improved by integrating MOFs with single-layer graphene constructs [74]. An MOF-based advanced chemical capacitive sensor for sensing ammonia at room temperature was developed by Assen et al. [75]. A rare-earth MOF thin film was deposited onto interdigitated electrodes (IDEs). The MOF-based gas sensor was initially trained for detecting ammonia levels in simulated breath systems in the presence of other interfering species such as carbon dioxide and humidity. Various functional groups can be integrated into the COF network, which allows for covalent immobilization of bioreceptors on the surface of transducers.

For example, NH_2_/COOH-functionalized COFs could allow the immobilization of DNA and proteins/antibodies through stable conjugation. Although the challenges with electrical conductivity limit the applications of COFs in electrochemical sensing, integration with other suitable nanostructures could widen the use of COFs in electrochemical sensors. An electrochemical sensor for detecting heavy-metal ions (Pb2+) was developed based on COF-modified electrodes [76]. The COF-based sensor detected the metal ion with excellent sensitivity and demonstrated the feasibility of the COF-based platform for electroanalytical measurement. The applications of AI have provided nanomaterial-based biosensors with new avenues in biomedical monitoring, clinical diagnosis, and high-throughput screening. In developing unique nano-systems, AI has also been used to investigate the properties of novel materials and their prospects in various fields. For instance, the turf morphology of CNTs can be determined by quantifying structural features such as curvature and alignment. Integrating AI with nanotechnology for designing novel nanomaterials with unique properties has crucial applications in targeted drug therapy (nanomedicine) [77]. The interaction of nanomedicine with biological systems such as blood and the cellular membrane is challenging in real environments and [78] can be easily simulated using machine algorithms. ML algorithms can be used to predict the drug encapsulation efficiency and cytotoxicity effects of nanoparticle formulations. Several computational models have been reported to predict nanoparticles’ ability to permeate across the blood–brain barrier (BBB). However, these models could help predict and develop novel nanomaterials with the ability to cross the BBB without affecting and altering biological functions. However, a detailed understanding of BBB permeation is essential but still a challenging task to achieve. Understanding nano–bio interactions with device biocompatible interfaces is crucial in healthcare applications, especially for developing implantable [79] or wearable sensors [80].

ML approaches have been widely explored for predicting the cytotoxicity of nanomaterials, identifying new non-toxic nanoparticles, and studying quantum mechanical electron motion for nano-electronics. ML has been used to predict the reactivity of chemical reactions and analyze faster than the manual methods [81]. Oh et al. reported a process for analyzing the cellular toxicity of Cd-containing quantum dots [82]. It is well known that nanoparticles’ cytotoxicity depends on physicochemical properties such as surface charge, core/shell architecture, size, shape, nature of surface ligands, exposure time, and exposure concentrations [83,84,85]. Oh et al. used advanced ML techniques such as the random forest method for mining and knowledge extraction from literature data to develop robust data-driven models for quantum-dot toxicity. Based on mining more than 300 publications and generating around 1700 quantum-dot cytotoxicity data, they predicted that the size, shell, and surface ligands, among the other parameters, influence the toxicity of the Cd-containing quantum dots. This research paves the way for understanding the toxicity mechanism of nanomaterials and helps in designing nanomaterials that are non-toxic in nature.

**Figure 7:**
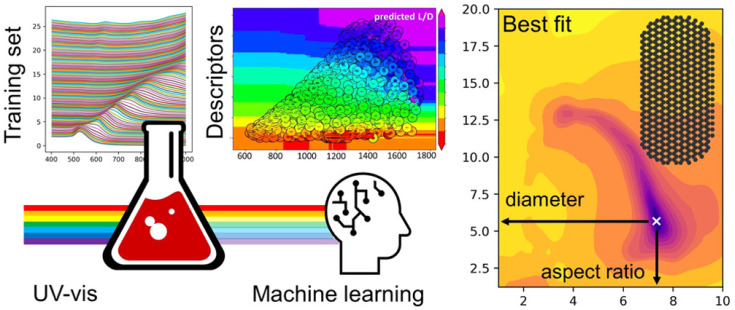
ML-assisted quantitative analysis of optical spectra of gold nanoparticles (Reproduced with permission from the American Chemical Society) [87].

ML approaches have also helped elucidate quantitative information from optical spectroscopic methods such as UV-visible spectroscopy (Figure 7). Au-NPs exhibit strong oscillations known as surface plasmon resonance (SPR). The SPR of Au-NPs depends on the size, shape, and surface modification. Based on the size, shape, and aspect ratio (gold nanorods), spectral properties such as bandwidth and position of the SPR can be determined [86]. Pashkov et al. used direct and inverse ML approaches for training and elucidating the complex dependencies in the SPR spectra of Au-NPs and structural properties [87]. They trained the ML algorithm to predict SPR spectra for the given structural parameters and the structural parameters for the given spectral parameters. Evolutionary algorithms find applications in the discovery and optimization of new nanostructured material. Researchers have used genetic algorithms, a sub-class of evolutionary algorithms, to speed up material development. In conventional research, the self-assembly of single-stranded DNA molecules onto colloidal particles was performed by allowing the DNA molecules to self-assemble without affecting the kinetics of the reactions. In this case, the crystal structure is examined after forming the self-assembled structure. However, Srinivasan et al. developed a methodology and genetic algorithm to design the self-assembly of single-stranded DNA molecules onto a desired structure [88]. The proposed genetic algorithm aided in developing new materials for creating self-assembled structures and helped mitigate the issues associated with time-consuming conventional trial-and-error methods.

In the same direction, ML approaches are also used to create new engineered bioreceptor molecules for intelligent sensing applications. Predicting the recognition ability of the receptors in a severe environment is crucial for improving the selectivity and specificity of the assay. Researchers have used deep neural network (DNN) algorithms to design and analyze a programmable RNA switch. The model developed could help understand synthetic bioreceptor switching behavior (ON and OFF state) [89]. Metallic glasses play a crucial role in developing magnetoelastic biosensors due to their unique combination of magnetostriction and soft magnetic properties. Ren et al. utilized ML iteratively with high throughput experimental methods to identify new metallic glasses [90]. The traditional search methods for metallic glasses involve the use of empirical rules, which are expensive and slow. They coupled the supervised ML approach, accelerated parallel synthesis, and high-throughput characterization to synthesize novel metallic glasses. ML approaches also help invent new flexible electronic materials for wearable sensing applications. Jackson et al. reported an ANN-electronic coarse graining ML approach for understanding the conformationally dependent electronic structures in soft materials [91]. Understanding semiconducting materials’ molecular structure and electronic arrangement has applications in developing high-performance optoelectronic devices. Supervised ML approaches have been used to compute semiconducting materials’ electronic structures quantitatively. The proposed method also has implications for various polymer-based devices and protein science.

Nano-enabled sensing strategies and AI-supported prediction with IoT platforms efficiently predict chronic diseases in very early periods. Nanomaterials play a crucial role in developing IoMT devices with better performance in biomolecule recognition, and the AI-assisted platform plays a crucial role in (i) the collection and transportation of raw data, (ii) processing the data that has been transported, and (iii) making a decision based on the data [92] The role of nanotechnology in improving the safety and efficacy of next-generation biomedical systems such as CRISPR/Cas9 gene-editing tools has also been reported [79]. Song et al. proposed that IoT sensors combined with AI analysis have a broad spectrum with intellectual transmission and great processing ability for healthcare workers during COVID-19 [93]. POC-sensing devices coupled with IoT and AI (such as machine learning and deep learning) have proven to help store and analyze data [40]. IMoT devices such as smart fabrics can detect blood pressure, heart rate, electrocardiogram, and body temperature. Commercial IoMT fitness tracking devices integrated with AI/machine learning, such as smartwatches and wrist bands, are getting greater attention in recent years due to the combined features of sensing and wireless data transmission [94]. Diagnosing primary communicable and non-communicable infections can be performed through e-diagnosing. This approach increases the diagnosing rate in remote areas with lesser cost [67,95].

## 4. AI-Supported Cardiac Monitoring

Cardiovascular diseases (CVD), the primary cause of mortality worldwide, caused nearly 18 million causalities globally in 2019 alone. CVDs are becoming a paramount concern, especially in low- and middle-income countries, due to their alarming cause of morbidity. Along with the genetic factors, lifestyle and stress alteration become significant risk factors for human CVDs. Based on the clinical conditions affecting the heart’s function, CVDs can be classified into various types, such as heart failure, heart attack, myocardial infarctions, cardiac arrhythmia, pericarditis, and cardiomyopathy. Different tools are available for monitoring irregularities in heart function, including cardiac computed tomography (CT) scan, electrocardiogram (ECG), Holter monitoring device, stress test, and blood biomarker profiling. Among them, ECG, stress test, and biomarker profiling provide a rapid tool for cost-effective assessment of heart function. Stress is also one of the main reasons for myocardial infarctions. Physiological stress is also one of the critical tasks, which can be monitored in a personalized manner depending on the lifestyle, situations, and circumstances using a biosensor. Electrochemical biosensing devices allow for stress measurement by estimating cortisol [72,96,97], a physiological stress biomarker [96].

This review section summarizes the recent developments in rapid diagnosing tools for accurately detecting various pathological conditions associated with CVDs. Atrial fibrillation (AF) is a condition of having an irregular heartbeat, and the population with AF is prone to developing blood clots, which could lead to cardiovascular malfunction and even cause stroke. A diagnosis challenge arises here because AF is asymptomatic and remains undetected until the first thromboembolic event occurs. A thromboembolic event is related to forming a blood clot in the blood vessel, which the bloodstream can carry to block another blood vessel. Clinical trials such as the Embrace trial (a 30-day screening in patients with cryptogenic stroke) and crystal AF trial (a 36-day study of continuous cardiac monitoring to assess AF after cryptogenic stroke) have been performed for long-term continuous monitoring of AF. However, as mentioned earlier, the trials have bottlenecks for all suspected AF because of the inconvenience in usage, lack of reimbursement, and other technical reasons. For long term-monitoring of AF, the method should be reliable, cost-effective, convenient, and with easy-to-apply tools for extended non-invasive AF detection.

**Figure 8:**
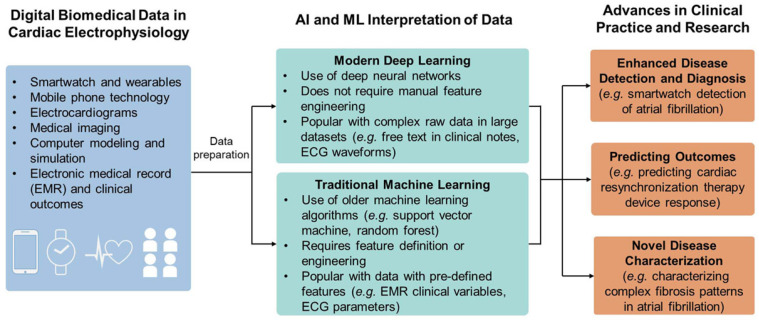
Role of AI/ML in cardiology. The biomedical data collected through cardiac electrophysiology measurement are interpreted through either traditional or modern ML algorithms for advancing the health outcome.

Electrocardiogram (ECG) is one of the contemporary methods for real-time monitoring of cardiovascular function. To predict various cardiac diseases, ECG is useful as it can provide the morphological and functional details of the heart. Irregularity in the heart rhythm is conventionally monitored using a 12-lead ECG recorder. However, manual interrogation of ECG recording is time-consuming, and analysis of a large volume of data may cause errors. Indeed, the conventional ECG measurement tool causes inconvenience to the wearer and is prone to noise during ECG measurement. In recent years, single-channel ECG recorders have evolved as sensitive devices for accurately detecting variation in the heartbeat. However, the data produced by the single-channel ECG recorder are enormous and require an automated program to process the large volume of data sets and evaluate the measurement’s specificity. Researchers have used ML-based algorithms to diagnose arrhythmic heartbeats and predict abnormalities accurately. From the ECG data, the characteristic features extracted can be employed to detect cardiac-related conditions such as myocardial infarctions, sinus tachycardia, and sleep apnea [98]. Through advancements in cloud computing and capabilities to process a large set of data, AI/ML has shown promise in monitoring cardiac electrophysiology and cardiac imaging. AI (DL/ML)-based systems have been explored for various applications, including analyzing ECG signals for noise classification, arrhythmia identification, prediction of atrial fibrillation, and analyzing whole-genome sequences. An overview of the role of AI/ML in electrophysiological measurement is provided in Figure 8. The data acquired from IoMT devices such as smart watches, mobile phone technologies, and medical imaging are trained and processed by AI/ML for enhanced disease diagnosis, predicting outcomes, and characterizing novel diseases.

Kachuee et al. proposed a novel framework deep learning algorithm for the analysis of ECG data that can represent the signal in a convenient form for evaluating different tasks such as ECG signal recognition and heartbeat irregularity identification [99]. The convolutional deep neural network algorithm is trained with Physionet MIT-BIH arrhythmia and the PTB diagnostic database. AI preprocesses ECG signals before they are used as input for heartbeat classification. A deep convolution network was used to classify the ECG heartbeat type and training prediction task. Then the tensor flow library was used for model training and evaluation. The trained deep convolution neural network was used to evaluate 4079 heartbeats for evaluating arrhythmia. However, the group reported that an exact predictor for MIT-BIH datasets is not proposed, but that the planned method excelled in accuracy compared to state-of-the-art methods. Photoplethysmograph (PPG)-based smartphones are used for screening AF. Smartwatches with PPG sensors are the new-generation methods adopted for detecting AF. Dörr et al. proposed the WATCH AF trial, comparing the diagnostic accuracy to detect AF by a smartwatch-based PPG algorithm using PPG signals with cardiologists’ diagnosis by ECG [100]. The smartwatch’s (Gear Fit 2) integrated PPG sensor recorded the PPG data collected by the Samsung SE mini smartphone and transferred them to the server. A SINGLE-LEAD iECG (Alivecor Kardic Systems) was connected to an iPhone 4s and ECG data were collected and saved as a PDF. The second PPG recording was on another wrist by a smart band (wavelet wristband) connected to an Apple iPad mini, and the data were transferred to the server. PPG signals were extracted as 1, 3 and 5 min segments and proposed by an automated PPG algorithm.

Based on the noise-to-signal ratio, 1 min segment data were taken for final analysis. By comparing the PPG algorithm-based diagnosis with cardiologists’ interpretation of iECG, it was found that the novel PPG algorithm-based diagnosis was effective in terms of an overall accuracy of 92%, a sensitivity of 98%, a selectivity of 93.7%, and better PPV (positively predicted vale) and NPV (negative predicted value). The main limitation of the model is only comparing it with iECG data but not with standard 12-lead ECG and Holter ECG. PPG-based AF detection devices may still be understood as AF screening tools with a need for a confirmatory ECG for suspected AF. Li et al. studied genomes for identifying abdominal aortic aneurysm (AAA) using an ML framework from personal genomes and electronic health records (EHR). This usually results in the effect of personal genomes and individualized lifestyles. For the first time, they introduced high-coverage whole-genome sequencing (WGS) for AAA patients with the help of HEAL (hierarchical estimate from agnostic learning). HEAL is a subset that identifies the distinct patterns in the genomes and then uses those patterns to identify the outcomes. This subset even identifies the outcomes at the mutation level. This is based on the framework of hierarchically estimating and agnostically learning [101].

## 5. Role of AI in Surgery

The significance of IoMT is rapidly increasing due to the combined growth of AI and its several subsets, like ML, computer vision (CV), deep learning (DL), and natural language processing (NLP). All of the aspects of AI stated above give a basis for all the autonomous actions in AI-assisted surgeries [102]. The rapidly growing capabilities of AI in fields like surgeries can be attributed to the combined inputs and outputs of the subfields of AI. AI is applicable at various levels of surgery. Collective data on applications of AI in different spinal surgeries have been summarized by Chang et al. [27]. The development of automation in surgeries has led to the increased use of AI. In traditional surgeries, humans used to perform all the roles. The surgeries became completely AI-based and autonomous over time, from partial roles like image guidance to operations where no direct human involvement is required. This has been discussed by Panesar et al. [103]. Da Vinci Surgical System is one of the well-known robotic-assisted surgery systems. The surgical system allows the doctors to perform surgery from a remote booth with equipped technologies to control the arms of the robot [104]. This is a minimally invasive method, and is usually a trusted method by most physicians due to its accuracy and innovation. Panesar and Ashkan et al. discussed the role of the internet or mobile platforms, which are controlled by AI and can be used to provide surgical expertise remotely. It may be used to guide a surgical robot to perform surgeries where appropriate resources are unavailable or access is lacking, such as a spacecraft in space or places with environmental disaster or war [105].

### Role of AI in Spine, Cardiac, and Eye Surgeries

**Figure 9:**
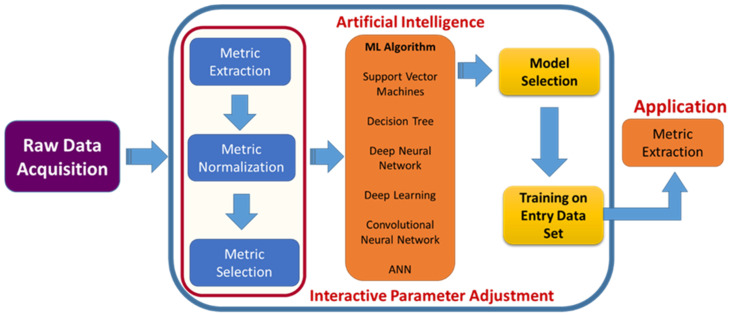
Role of AI in surgery. Framework integrating AI in spinal surgeries, which involves raw data acquisition to convert the inputs into digitalized form and pre-processing methods for machine learning such as metric extraction to train the ML and metric selection to differentiate between two groups. The optimum algorithm is selected based on the input, and then the output is generated.

Usually, surgeries are of many types, and some may be scheduled. It does not mean that it could be optional but that it can be scheduled per convenience. The other type is emergency and could be life-threatening depending on medical conditions. Similarly, there are four crucial primary objectives in spinal surgery care, which include (i) preoperative duties, selection of patients based on their level of disease, and prediction of results after surgery; (ii) enhancing the quality and reproducibility of spinal research; (iii) data collection and tracking before the surgery; and (iv) intraoperative surgical performance [104]. This massive increase in artificial intelligence with machine learning opens up a new way for surgeons to analyze the data more precisely [26]. AI accompanied by ML is used for diagnostic spinal imaging, the prediction of therapeutic interventions, information retrieval, biomechanical analysis, and the characterization of biological tissues (Figure 9).

Devoid of the type of surgeries, AI facilitates surgeries in many ways. The annual expenses for spinal care in the USA are around USD 110 billion and are expected to reach up to USD 5.3 trillion by 2025 (Rasouli et al., 2021). In addition to this, there is a huge difference in the opinions, delivery of services, care, and the costs of surgeries among different countries or even among different hospitals in the same country. Ames et al. suggested that the use of AI in these surgical treatments may help to define the quality and expenses of treatments and care. It can also improve the results and lower the expenses of patients as well as hospitals [106]. There are several models proposed to allow patients and surgeons to predict the risks involved with surgeries. A logistic regression model was used in the assessment of risk by Bekelis et al. [107]. Sheer et al. used a decision tree for complication predictions in adult spinal deformity surgeries [108].

AI also helps gather and process diverse information such as risks involved, the anatomical information, genetics and other histories of the disease, and economics of patients, and to make better predictions of surgeries [109]. For example, in some epileptic patients, it can be predicted by a DL model, due to which patients could better benefit from surgeries. AI can provide directions to surgeons in the operating room so that the surgery can be performed with minimal risks. Previous studies have developed several machine learning algorithms in cardiothoracic surgeries, which can beat standard operative risk scores in predicting postoperative deaths in cardiac patients [110]. Ostberg et al. discussed that ML-based methods like artificial neural networks and convolutional neural networks had been used in studies such as segmentation of ascending and descending aorta and detection of common chest X-ray pathologies, detection of wall motion abnormalities on echocardiograms, and segmenting the left ventricle to continuously measure ejection fraction in cardiac/thoracic surgeries [111]. Li et al. discussed an ML approach integrated with genomic and electronic health record data, confirming a substantial ability to study abdominal aortic aneurysms and underlying genetic mechanisms [101]. Cardiovascular surgeries also have the risk of acute kidney injury associated with them. The mortality rates are 10.5% and 30%, in the case of cardiac surgeries and acute kidney injuries, respectively, which increase with the severity of kidney injury. Various machine learning methods help predict the postoperative risks of cardiac surgery-associated kidney injuries. Models such as logistic regression, simple decision tree, random forest, support vector machine, extreme gradient boosting, and ensemble have shown promise and helped minimize postoperative complications [98].

**Figure 10:**
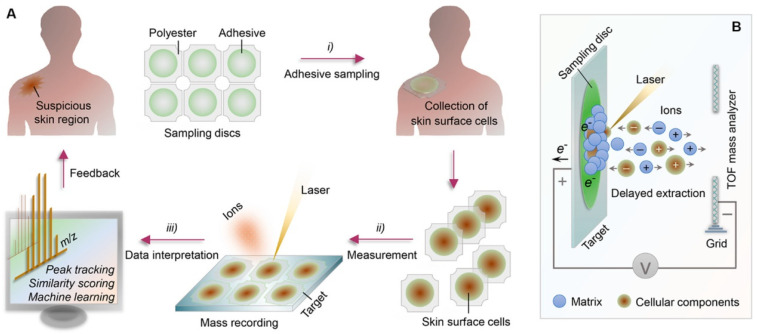
(**A**) Schematic representation of the methodology used for skin diagnosing. (**B**) Representation of matrix-assisted laser desorption ionization time-of-flight (MALDI-TOF) mass spectroscopy.

Phillips et al. performed an assessment on an AI algorithm to detect melanoma in images of skin lesions [112]. While comparing other skin cancer types, malignant melanoma is uncommon. It should be diagnosed in the early stage and be monitored regularly. A cancer diagnosis in Stage I has a 95% relative survival rate compared to a late diagnosis in stage IV, with a relative survival rate of 8% to 25%. The algorithm used here is the DL process. Zhu et al. developed an analytical chemical methodology to achieve rapid, non-invasive, and high-throughput skin monitoring [113]. For recording the skin-surface mass profile, an adhesive sampling procedure is combined with matrix-assisted laser desorption ionization time-of-flight (MALDI-TOF) mass spectroscopy. Mild cell samples are collected using adhesive sampling methods from epidermal skin layers. MALDI-TOF mass spectroscopy is implemented in chemical laboratories, and results are obtained in minutes by detecting analytes based on their molecular weight (Figure 10). At a time, several samples can be placed and analyzed. The mass spectral can be easily analyzed because most signals are due to the singly charged analyte ion. Using AI such as ML, data mining, or complex network analysis for automated data interpretations enables us to process extensive complex data quickly. Nevertheless, such work is still at the testing level and has not been applied to human skin yet, but it has been successfully tested in mice and produced good results.

## 6. Role of AI in Diabetes Mellitus and Cancer Management

Diabetes is one of the rising concerns in healthcare and making it essential for periodic monitoring of blood-glucose levels. Measuring blood glucose is one of the crucial measures for people with hypoglycemia and hyperglycemia. About 425 million people globally are in the hands of diabetes, and ~12% of the world’s total expenditure is spent on diabetes management. The estimated expenditure on diabetes is expected to increase to USD ~490 billion by 2030. Chronic diabetes could lead to diabetic retinopathy (DR), which causes partial or complete blindness [114]. According to epidemiological studies (i.e., the study of frequency and the potential causes of the disease), one in three diabetic persons suffers from DR, and the third one suffers from diabetic maculae oedema, which are tiny bulges that protrude from the walls of vessels, which then leak blood into the retina and cause severe health concerns [14]. Accuracy and frequent glucose monitoring are necessary to prevent both acute and chronic clinical impediments caused by diabetes. As the conventional glucose-monitoring technique requires puncturing the skin and drawing blood, it is essential to develop a technology for patients at an affordable cost without pricking their fingers multiple times to check the glucose level. Currently available glucose-monitoring systems for POC measurement in patients are based on electrochemical approaches. Although some shortcomings are present regarding the accuracy and precision of glucometers, the usage of glucometers for POC diabetes management is increasing every year. Whereas point-of-use glucose meters provide a snapshot of glucose trends, a continuous glucose-monitoring system (CGM) provides real-time information on glucose levels to both the patient and the caregiver. The complexity of blood dynamics is one of the significant challenges for accurate and early prediction of glucose levels. Methods based on AI/ML, natural language processing, and artificial neural networks are highly significant in controlling diabetes, as they help predicting diabetes patterns and diagnose the risk of diabetes, which makes diabetes management easy [115].

**Figure 11:**
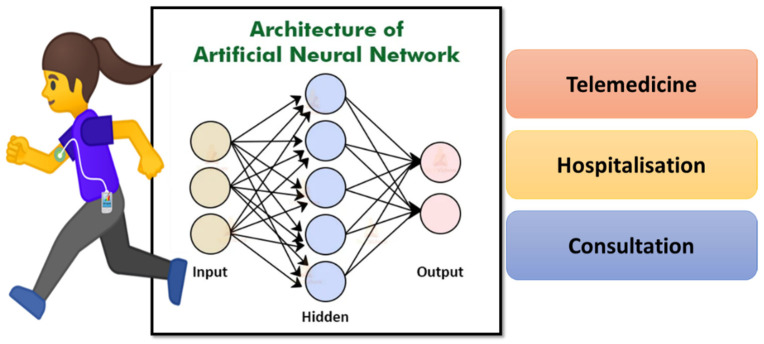
Representation of artificial neural network using patient data to identify the correct treatment plan.

AI- and ML-based approaches have been incorporated with glucose-monitoring devices to improve clinical accuracy. For example, Khanam et al. developed an AI-enabled system for accurately detecting human glucose levels [38]. Five input features such as age, pregnancy, body mass index, glucose, and insulin levels are used to train the system. ML algorithms such as RF, NN, DT, and K-nearest neighbor (KNN) with hidden layers were used to evaluate the data set. All models yielded a reliable measure of glucose with an accuracy of >70%. Hamdi et al. analyzed the level of glucose using a hybrid system with compartmental models [116]. The glucose levels were monitored using the CGM method with the help of the ANN algorithm. The device consists of a subcutaneous sensor placed just under the skin connected to the transmitter, which is further attached to a wireless receiver to display the glucose levels. The device measures glucose levels every 15 min. To evaluate the system’s function in clinical analysis, the data of 12 patients were collected for training and validation steps. This algorithm consists of three layers: an input layer, a hidden layer, and an output layer (Figure 11).

**Figure 12:**
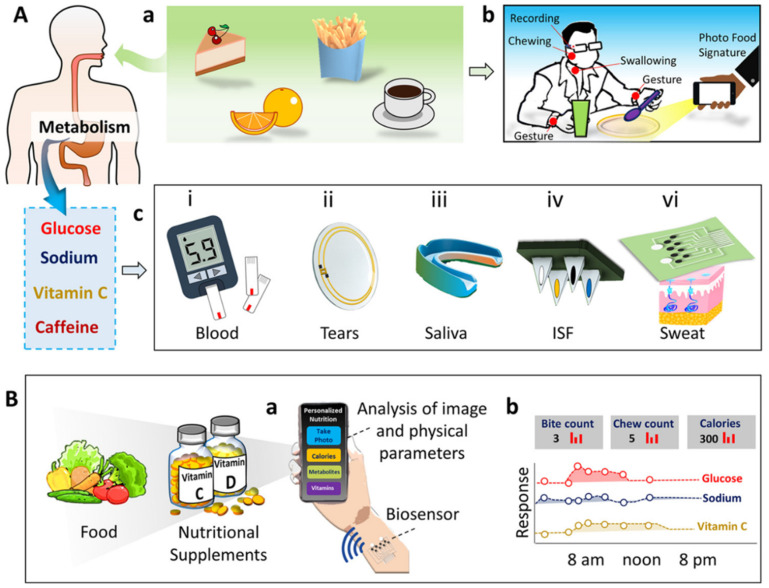
Concept of personalized nutrition measurement system. (**A**) Monitoring food intake (**a**) and ingestion behavior (**b**). Wearable sensing of metabolites in human biofluid (**c**). (**B**) Schematic representation of comprehensive nutrient-monitoring system for simultaneous monitoring of nutrients present in the food and metabolites in humans. (Reproduced with permissions from the American Chemical Society) [118].

Rigla et al. monitored gestational diabetes with a group of 247 patients using a decision-support system through telemedicine during the COVID-19 pandemic [117]. The monitoring system consists of a smartphone for information on physical activities and a glucometer with Bluetooth for data transfer. The collected data from the hospital electronic medical report (EMR), blood-pressure monitor, and glucometer are fed to the decision support system (DSS) algorithm as an input for training. The ML algorithm is programmed to suggest a diet plan for the patient and indicates to the patient whether a doctor visit is needed. Personalized precision nutrition is the concept of individualizing the nutrition/diet plan based on metabolism rate, gender, age, and biochemistry for treating, managing, and preventing diseases. Integrated biosensor devices enable the development of personalized nutrient development approaches by monitoring specific biochemical molecules. Wang et al. recently proposed the concept of personalized nutrition monitoring by integrating bio-sensing devices with cloud-based systems [118]. The proposed multimodal sensing platform monitors food intake and ingestion through imaging and motion sensors. The system also uses wearable sensors to measure metabolites and nutrients in human biofluids such as blood, sweat, saliva, urine, and interstitial fluid (ISF) (Figure 12)**.**

Marcus et al. developed a personalized SML approach for glucose-level prediction. The SML techniques allow for the identification of patterns and relationships in the data sets, which are non-linear. Addressing non-linearity is essential in addressing the glucose sensors’ clinical accuracy. Data from 11 volunteers were collected to train the system. The CGM devices utilized interstitial fluid (ISF) to assess glucose concentrations. However, the time lag between glucose changes in both compartments is reported as 5 to 25 min. Because of this time delay, automated insulin injections (artificial pancreas) are not recommended for CGMs. Notably, the CGM often fails to identify hypoglycemic events on time. The algorithm developed by this team predicts elevations in sugar levels 30 min in advance, enabling the implementation of artificial pancreas systems in CGM [119].

Non-invasive glucose sensing is an emerging area for continuous glucose monitoring and has the potential to replace the conventional finger-prick-sensing approach. ML-based optical sensors for monitoring glucose levels [24] using different wavelengths of light sources have been demonstrated. More than 21 different light sources with varying wavelengths were used, and five different ML approaches have been implemented for glucose analysis and prediction. The prediction accuracy of the system was improved by arranging the data sets into 21 classes. The system showed good capability in discriminating between higher, lower, and normal glucose levels. Paper-based analytical devices (µPADs) integrated with a smartphone for colorimetric sensing of glucose levels in saliva have been reported for POC measurement [120]. Three different combinations of chromogenic agents were tested for producing color when glucose reacts on the work surface of µPADs. The images were acquired in four different smartphones in seven different illumination conditions. Various ML classifiers were screened, and the best machine classifiers for each detection condition were optimized to enhance the detection accuracy. The data sets were then processed using a cloud-based system that controls the classifier remotely.

Cancer is the clinical condition of unmanageable growth and spread of atypical cells throughout the body. Imaging tests used to spot cancer are computerized tomography (CT), magnetic resonance imaging (MRI), positron emission tomography (PET) scan, and ultrasound. Biomarker-based detection strategies have also been developed to detect FDA-approved protein cancer markers early [121,122]. AI-based technologies have potential applications in cancer research, including early detection, screening in a large population, classification and stage grading, molecular characterization, prediction of patient outcomes, treatment responses, personalized treatment, automated radiotherapy workflow, and novel anti-cancer drug discovery and clinical trials. AI and ML algorithms can also be used to create prediction models for assessing lymph node metastases, response to drug treatments, and prognosis. Using clinical data, pathological data, and genetic polymorphisms in an ANN model, researchers could predict the preoperative stage of stomach cancer with an accuracy of 82% [123]. Many researchers have used AI-based algorithms to develop computational models to predict cancer outcomes. Gene expression proved an efficient method, but it comes with the drawback of limited sample sizes. The AI-coupled ML system has proven to be efficient in detection, diagnosis, and subtype classification [79]. The ML algorithms have been recognized as a preferred alternate tool for pattern recognition in breast cancer [51]. Decision-prediction algorithms, including K-nearest neighbor (KNN), support vector machine (SVM), and decision tree (DT), aid in extracting the clinical features from the overcrowded datasets. Fan et al. developed an AI-aided 3,3′-diaminobenzidine (DAB)-based immunohistochemical method to detect multi-tumor [124]. Based on the proposed model, the group diagnosed HER2 overexpressed breast cancer with high sensitivity (95%) and selectivity (100%). AI aided immunohistochemical method to detect the multi-tumor, which overcomes the sensitivity limitation of the manual immunohistochemical method.

## 7. Challenge and Future Prospects

**Figure 13:**
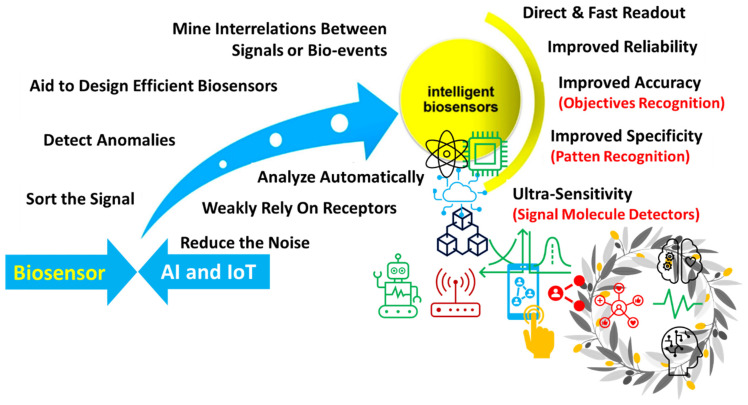
Role of AI/ML in advancing the performance of biosensor systems. (Reproduced with permission from the American Chemical Society) [125].

The advantages of AI in diverse healthcare sectors, such as monitoring cardiac arrhythmia, diabetes management, and assisted surgeries, have been reported. ML aids in processing extensive and complex sensor data effectively for further analysis and improving decision-making abilities. AI/ML also helps extract the analytical data from low-resolution or noisy data sets. Through SML approaches, the AI/ML technique allows the IoMT devices to extract the hidden information based on the relationship between the sample parameters and measured signals. The AI techniques also improve the signal strength, sensitivity, specificity, and measurement time (Figure 13).

Although AI/ML has shown the potential to revolutionize the healthcare practice and IoMT-integrated medical devices, numerous technological challenges need to be addressed to realize the prospect of commercialization and adaptability at clinics and in society. As the AI/ML systems rely heavily on accurate data for programming and training the system, the focus must be on collecting extensive data on quality patient training and learning. Another critical challenge is heterogeneity in collected data. The health records collected from different clinics have various types of bias and noise, which cause discrepancies in AI training. Sophisticated ML algorithms can help homogenize the data sets to improve the accuracy of the clinical diagnosis. With AI-supported techniques, the future of surgery and the medical field will bloom.

The connectivity of technologies is vital in connecting people with IoMT devices. The connectivity can be unidirectional or bidirectional. The IoMT sensor signal must be processed before being sent to the microcontroller/processor, which requires only digital data. The analog front end (AFEs) eliminates the bulk electronics required for signal conditioning. The connectivity between AFE and the microcontroller is usually established by communication protocols such as I2C, SPI, and UART. There should be compatible communication protocols between AFE and the microcontroller. Wi-Fi and Bluetooth are the main connectivity methods available for interfacing IoMT devices with the central hub. Bluetooth connectivity is suitable for short-range communications up to 10 m with a maximum speed of 3 Mbps, which connects sensors to various portable gadgets such as tablets, smartphones, and PCs. Data transmission through Bluetooth is suitable primarily within the operating room, ICUs, and other locations with more devices. Enterprise Wi-Fi is used for connecting the IoMT devices to the gateway, which provides a higher level of service when it comes to security and performance. When the measuring device is in motion, Internet connectivity could be lost, which could lead to a loss in critical data and delay healthcare administration to a patient. Thus, the Wi-Fi module must support optimized scanning algorithms to maintain network persistence for the mobile devices within these noisy RF environments.

Conventional medical devices for diagnostics require complicated and bulk electronic components to compensate for electrical and physical errors. For instance, optical devices need to define the optical path and ambient light interference elimination, which require a complicated enclosure design. Optical devices designed for portable applications always need to detect low-yield fluorescence while at the same time rejecting system noise. Although conventional electrochemical biosensors require electrochemical workstations, which are bulky and expensive, the POC or wearable biosensor devices require portable electronics. The bulk electronics required can be replaced with single IC solutions, also known as AFEs. Most AFEs are available as a separate package for distinct types of sensors. For instance, AD5940 from Analog Devices Inc. can only be used for electrochemical biosensors. This AFE has limitations with different multiplexing types of sensors, such as both optical and electrochemical sensors to a single AFE. The current generation of IoMT devices requires multi-functional AFEs with multiple channels for interfacing with an array of sensors. For example, the miniaturized potentiostat [126] (M-P), developed through customizing LMP91000, offers POC testing capabilities and provides low-power measurement [127,128,129] and high sensitivity. However, the multi-channel interfacing of M-P and further smartphone operation is still challenging but has good aspects.

The next-generation devices would also allow for efficient processing of the signal received for both noise reduction and data management. Mobility has opened new avenues for IoMT healthcare devices. The scope of mobility in IoMT has also grown with increased smartphone access. Healthcare providers are opting for smartphone-based mobile devices for user-friendly operation. Mobile health technologies enable telecommunication, bringing the caregiver and the patient together for cost-effective online consultation. IoMT devices equipped with mobile apps can control, access, monitor and track intelligent devices. Nevertheless, mobile devices face challenges with security and privacy issues. Healthcare providers are exploring innovative solutions to address security challenges and safe data transmission.

Another critical problem associated with AI is the management of extensive data. These data are usually called “big data.” This problem occurs when humongous inputs are given, and there is a high risk of system collapse in such conditions. Hence, there is a need for an intelligent and scalable machine learning algorithm to process such big data [130]. The other paramount concern is data privacy. All patients’ data are stored in the cloud, and there is a high chance of leaking and misusing such data [131]. Like any other medical treatment or device, AI-based devices or treatments also undergo stringent validation processes before entering clinical practices. The processes of validation and approvals vary according to intended uses and forms. The AI in healthcare is designed to perform one job at a time. The initial wave of effort is made to understand and resolve the backbreaking outputs. One main problem is the need for experts who can resolve the outputs that are complex for humans. As discussed above, AI is incapable of competing for multitasking. A system that can multitask is yet to be developed to overcome this issue. The influence of AI technologies on the healthcare sector is improving and likely to dominate in the next decade. Even with the recent advancements, replacing a physician’s diagnosing role with computers is still a long way away.

We are in the era of exploring the depth of AI/ML in healthcare diagnosis, which will thrive in IoMT systems and help us unlock new avenues in the healthcare sector. With the advancements in nanotechnology and microelectronics, AI-based IoMT devices will move in the direction of multi-level functionality, high sensitivity, industrial-level production, miniaturization, ultra-low power consumption, and inexpensiveness. With the continued integration of AI/ML systems with IoMT devices and integrated medicine, people will progressively access high-quality healthcare.

## 8. Conclusions and Viewpoint

This review reports on the recent developments and advancements made mainly in AI-supported IoMT devices for efficient biosensing needed for successful disease management. In support of aspects of AI and IoMT, we have discussed the significance of nanotechnology in the IoMT platform for developing next-generation biomedical devices such as e-skin, e-nose, and e-textiles. AI-integrated IoMT devices are important in crucial medical areas such as cardiac monitoring, surgeries, diabetes, and cancer monitoring. Through cloud computing, AI has shown promise in monitoring cardiac electrophysiology and imaging. The innovations made by AI in the surgery sector are noteworthy. One such remarkable invention is the Davinci surgical system, a robotic-assisted surgical system. AI interfacing established a quantum shift in diabetes and cancer management in a personalized manner.

AI-based outcomes support earlier prediction and determine the level of risk while diagnosing disease. Many physicians prefer ML algorithms for prediction because of their data accuracy. Besides ML, AI subsets such as SVM and NN are also widely used in the clinical sector. Every innovation has challenges, such that AI faces heterogeneity, connectivity, and extensive data management issues. The solutions for these problems have been discussed in this review. Apparently, AI cannot do multiple jobs at a time, and the complete replacement of a physician is still yet to be developed. Despite this shortcoming, AI has proven its excellence in the medical field with continuous evolvement. The outcome of this review motivates young researchers to promote and investigate combinational approaches involving nano-enabled sensing, AI, and IoMT for efficient biosensing needed for disease control and management in a personalized manner.

## Figures and Tables

**Table 1:** Comparison of applications along with advantages and disadvantages of SVMs, NNs and other common AI algorithms used in biomedical applications.

AI Algorithms	Applications in Medical Sciences	Advantages	Disadvantages
**Support Vector Machine (SVM)**	Biomarker imaging in neurological and psychiatric disorders [18] Human–machine interface [19] Cancer diagnosis [20] Early detection of Alzheimer’s disease [21] Cardiac monitoring [22] Predicting surgical site infection [23] Glucose monitoring [24] Surgery [25,26,27] Pandemic resource management [28] Healthcare monitoring system [29]	Highly accurate, convergence to a solution for a problem is faster, solving complex problems, good scaling for high-dimensional data, and requirement of a minimum number of training samples.	Selecting appropriate kernel function is important, requirement of longer training time for large datasets, high computational cost. Difficulties in understanding and interpreting the final model, variable weights, and individual impacts. Problems in managing the missing values and prone to overfitting.
**Neural Network (NN)**	Cancer diagnosis [13,30,31,32] Identifying Parkinson’s disease [33] Image-based cardiac monitoring [22] Alzheimer’s disease [34,35] Surgery [25,26,27] Sensor applications [36,37] Diabetes prediction [38] Human–machine interface [39] Pandemic resource management [40] Computer vision [41]	Efficient, fast, and flexible algorithm. Calculates output without programmed rules, continuously learns and improves itself. Multitasking and has wide applications. It can work with nonlinear and complex databases.	Longer training time and large datasets are required. High hardware cost and requires lengthy and complex programs. Interpretation and modification are difficult due to black box nature. Prone to overfitting. High data dependency may give faulty results.
**Naïve Bayes (NB)**	Disease prediction [42] Medical diagnosis [43,44] Systems performance management [44] Pandemic resource management [29]	Easy implementation, high learning and classification speed. Capable of managing overfitting, noisy data, and missing values. Able to predict the class of a test dataset. Useful for solving multi-class prediction problems.	Biased for non-ideal training set. Challenges in performing regression and co-dependent features. Not suitable for complex problems.
**K-Nearest Neighbor (KNN)**	Glucose monitoring for diabetes [24] Pandemic resource management [28] Disease prediction [45] Computer-aided diagnosis [46] Heart-disease prediction [47] Healthcare-monitoring system [29]	Simple algorithm. No assumptions for features and output of the dataset. Effective against noisy data, managing large data. Stable performance, high learning speed, and good overfitting management.	Time expensive, sensitive to local data. Moderate accuracy, slow classification speed. Poor handling of correlated data
**Decision Tree (DT)**	Glucose monitoring for diabetes [24] Surgery [26,27] Medical diagnosis [44] Systems performance management [44] Healthcare-monitoring system [29]	Very fast, efficient, and simple to understand and interpret. Can handle a large variety of data types. High computational, learning, and classification speed.	Complex calculations. Time and computationally expensive. Poor in handling overfitting, noisy, and correlated data. Inadequate at performing regression and has medium accuracy.
**Random Forest (RF)**	Disease prediction [48,49] Healthcare-monitoring system [29] Heart-disease prediction [22]	Good for managing noisy data. High classification speed. Good for handling large and heterogeneous databases. Automatic feature definition. Input feature normalization is not required.	Complex work function, difficulties in implementation. Moderate accuracy, slow learning speed, poor handling of missing values. Prone to overfitting. Proper definition of depth and number of trees is important.
**Logistic** **Regression**	Image-based cardiac monitoring [22] Glucose monitoring for diabetes [24] Pandemic resource management [28] Healthcare-monitoring system [29,50]	Simple implementation and interpretation. Good training efficiency. Outputs are well-calibrated and classified. Empirical parameter tuning is not required. Good accuracy for simple data sets.	Fails to solve non-linear problems. Assumes linearity in dependent and independent variables. Prone to overfitting for high-dimensional datasets. Highly dependent on parameters and features.
